# Essential role of a conserved aspartate for the enzymatic activity of plasmanylethanolamine desaturase

**DOI:** 10.1007/s00018-022-04238-w

**Published:** 2022-03-28

**Authors:** Ernst R. Werner, Monica L. Fernández-Quintero, Nicolas Hulo, Georg Golderer, Sabrina Sailer, Katharina Lackner, Gabriele Werner-Felmayer, Klaus R. Liedl, Katrin Watschinger

**Affiliations:** 1 Institute of Biological Chemistry, Biocenter, Medical University of Innsbruck, Innrain 80-82, 6020 Innsbruck, Austria; 2 Department of General, Inorganic and Theoretical Chemistry, University of Innsbruck, Innrain 80-82, 6020 Innsbruck, Austria; 3 Institute of Genetics and Genomics of Geneva, University of Geneva, 1, rue Michel Servet, 1211 Geneva 4, Switzerland

**Keywords:** Plasmalogen biosynthesis, Di-iron center, Predicted structure, Site-directed mutagenesis

## Abstract

Plasmalogens are an abundant class of glycerophospholipids in the mammalian body, with special occurrence in the brain and in immune cell membranes. Plasmanylethanolamine desaturase (PEDS1) is the final enzyme of plasmalogen biosynthesis, which introduces the characteristic 1-*O*-alk-1′-enyl double bond. The recent sequence identification of PEDS1 as transmembrane protein 189 showed that its protein sequence is related to a special class of plant desaturases (FAD4), with whom it shares a motif of 8 conserved histidines, which are essential for the enzymatic activity. In the present work, we wanted to gain more insight into the sequence–function relationship of this enzyme and mutated to alanine additional 28 amino acid residues of murine plasmanylethanolamine desaturase including those 20 residues, which are also totally conserved—in addition to the eight-histidine-motif—among the animal PEDS1 and plant FAD4 plant desaturases. We measured the enzymatic activity by transient transfection of tagged murine PEDS1 expression clones to a PEDS1-deficient human HAP1 cell line by monitoring of labeled plasmalogens formed from supplemented 1-*O*-pyrenedecyl-*sn*-glycerol in relation to recombinant protein expression. Surprisingly, only a single mutation, namely aspartate 100, led to a total loss of PEDS1 activity. The second strongest impact on enzymatic activity had mutation of phenylalanine 118, leaving only 6% residual activity. A structural model obtained by homology modelling to available structures of stearoyl-CoA reductase predicted that this aspartate 100 residue interacts with histidine 96, and phenylalanine 118 interacts with histidine 187, both being essential histidines assumed to be involved in the coordination of the di-metal center of the enzyme.

## Introduction

Plasmalogens are a special class of ether-linked glycerophospholipids, which contain a characteristic 1-*O*-alk-1′-enyl double bond (vinyl ether double bond). This feature is introduced by plasmanylethanolamine desaturase (PEDS1, EC 1.14.19.77). The enzymatic activity of this enzyme was already characterized in the 1970s [1]. Its gene and coding sequence has only been recently assigned independently by three groups [2–4]. The introduction of the characteristic 1-*O*-alk-1′-enyl double bond dramatically alters the chemical, biochemical and biophysical properties of this lipid class. The physiological importance of plasmalogens is still not fully understood [5, 6]. They are an important constituent of surfactants in the lung, and their destruction due to smoking is considered a mechanism of smoking-related lung diseases [7]. Low plasmalogen content in the brain was shown to be associated with severity of Alzheimer's disease [8], but it remains to be seen whether this is cause or consequence of the disease.

The recent sequence assignment of PEDS1 revealed occurrence of a conserved protein motif (pfam10520). This motif has also been observed in a special type of plant desaturases called fatty acid desaturase 4 (FAD4) which introduce a delta-3-trans double bond into phosphatidylglycerol in chloroplasts [9]. Membrane-bound lipid desaturases such as stearoyl-CoA desaturase have long been known to contain a characteristic conserved 8-histidine motif [10]. In the pfam10520 motif occurring in PEDS1 and FAD4 proteins, also a set of 8 conserved histidines can be found, but they are somewhat differently arranged [2, 3]. Site-directed mutagenesis showed that all of the 8 histidines conserved in both, FAD4 and PEDS1 proteins, are absolutely essential for catalysis [2, 3]. An additional histidine (H131 in mouse PEDS1) is only conserved in all PEDS1 but not in FAD4 proteins. When this histidine was mutated in the homologous position in CarF from *Myxococcus xanthus* and in human PEDS1/TMEM189 to alanine this yielded no response in the light response assay in *M. xanthus* [2]. A strongly reduced but still detectable formation of plasmalogens was observed when a murine PEDS1 protein with an alanine instead of a histidine at position 131 was introduced into PEDS1-deficient HAP1 cells [3].

Membrane-bound lipid desaturases are especially labile proteins which are difficult to be purified in active form. The PEDS1 homolog CarF from *M. xanthus* was purified, shown to have partial activity and to bind approximately two equivalents of iron [2]. Rare exceptions where enzymes of this class have been crystallized include stearoyl-CoA desaturase [11, 12] as well as the yeast homolog of fatty acid 2 hydroxylase [13]. The crystal structures confirmed the hypothesis that the set of conserved histidines binds the di-metal center. Although it had long been known that iron is the metal contained in the catalytically active purified proteins [14], the crystal structures contained zinc instead of iron, which could not be replaced by iron [11, 12]. Only when expressed in a mammalian cell system engineered to have increased uptake of iron, recombinant iron containing stearoyl-CoA desaturase could be purified and crystallized [15].

**Fig. 1:**
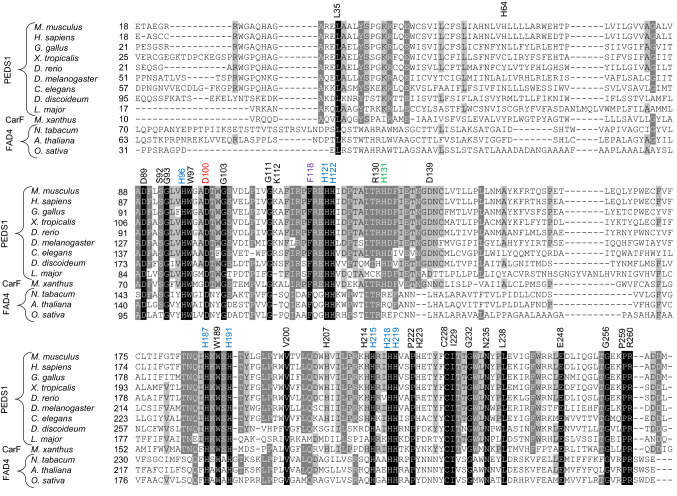
Alignment of protein sequences of PEDS1, CarF and plant FAD4 desaturases. PEDS1 sequences of selected species, CarF from *M. xanthus* [2], and FAD4 desaturases were aligned by Clustal W embedded in the MEGA X evolutionary sequence analysis package [16] and displayed using GeneDoc. Histidines labeled in blue had previously been shown to be essential for enzymatic activity [2, 3]. H131A labeled in green previously showed no activity in the respective CarF and human PEDS1/TMEM189 mutants in a light response assay in *M. xanthus* [2] but displayed residual activity by the HPLC/fluorescence method [3]. Histidine to alanine mutants in *M. xanthus* CarF and in human PEDS1/TMEM189 corresponding to murine H207, H214 and H223 were found to be active in the *M. xanthus* light response assay [2]. All other residues annotated were first investigated in this work for their impact on enzymatic activity, with the residues labeled red and magenta showing strongest effect of enzymatic activity when mutated (see below)

In addition to the mentioned set of 8 conserved histidines, 20 residues are totally conserved in a set of the most diverse FAD4 and PEDS1 desaturases (Fig. 1). Here, we mutated these residues in murine PEDS1 to characterize their importance for the enzymatic reaction. A few further residues not fully conserved were included as controls. Surprisingly, all but two of the mutants showed a residual activity above 20% of wildtype and only one of the mutations led to a total loss of the enzymatic activity. Using homology structural approaches, we constructed a predicted structure of murine PEDS1 which allowed a plausible explanation of these observations based on the predicted interaction of the two amino acids most important for the enzymatic activity with two essential, conserved histidines, respectively.

## Materials and methods

### Protein sequence alignment

Protein sequences of PEDS1 from selected species (accession numbers: *M. musculus*, NP_663513.1; *H. sapiens*, NP_954580.2; *G. gallus*, NP_001192029.1; *X. tropicalis*, NP_001192034.1; *D. rerio*, NP_001006052.1; *D. melanogaster*, NP_610026.1; *C. elegans*, NP_493036.1; *D. discoideum*, EAL72212.1; *L. Major*, CAJ03993.1), CarF [2] from *M. xanthus* (accession number WP_011555701.1) and FAD4 sequences from selected plants (accession numbers: *N. tabacum*, XP_016466208.1; *A. thaliana*, NP_194433.1; *O. sativa*, XP_015648482.1) were obtained from the protein database of the National Center for Biotechnology Information (NCBI) and aligned with the Clustal W program embedded in the MEGA-X evolutionary sequence analysis package [16], using default parameters. The alignment was displayed with GeneDoc with the similarity shading mode disabled (Nicholas Karl B., and Nicholas Hugh B., Jr, 1997, GeneDoc, a tool for editing and annotating multiple sequence alignments. Distributed by the author).

### Cell culture and preparation of plasmids

Human HAP1 cells with inactivated PEDS1 (TMEM189) (RRID:CVCL_XU44, Horizon discovery, Waterbeach, UK, obtained December 2018) were transiently transfected with a CMV-promotor-driven expression plasmid with the reading frame of murine PEDS1 containing a C-terminal 6xmyc tag in the pEXPR-IBA103 vector (IBA, Göttingen, Germany) or with pEGFP-N1 (Clontech, Mountain View, CA, USA) as a control. Due to far better expression results, the murine rather than the human reading frame for PEDS1 was used. Site-directed mutagenesis was introduced using the Quikchange II site-directed mutagenesis kit (Agilent, Waldbronn, Germany), and the mutations confirmed by sequencing of the plasmids (Microsynth, Balgach, Switzerland). Cells were cultivated in IMDM (GIBCO 12440-053 obtained via Fisher Scientific, Vienna, Austria) containing 10% (v/v) fetal bovine serum (F7524, Sigma, Vienna, Austria) at 37 °C in a humidified atmosphere with 5% CO_2_. No penicillin and no streptomycin were added. The fetal bovine serum used was not freed from lipids and thus may contain plasmalogens, explaining a low background plasmalogen level we previously observed in PEDS1-deficient HAP1 cells [3]. This posed no problem in the present work, however, since we fed a fluorescently labeled precursor to the cells (see below) and only quantified newly synthesized fluorescent plasmalogens. Any plasmalogen incorporated into the cells from the serum would be non-fluorescent and, therefore, not detected by the method we used here.

### Transfection of cells

Cells were transfected using Turbofectin (Origene, Herford, Germany) and cultivated for 24 h. Every transfection experiment included a negative control using transfection of the green fluorescent protein (GFP) and a non-mutated wildtype positive control. Transfection efficiency was assessed using fluorescence microscopy of PEDS1-deficient HAP1 cells transfected with GFP as follows. 48 h post transfection cell nuclei were counterstained with 1 µg/ml Hoechst 33342 (Sigma-Aldrich, Vienna, Austria) for 10 min at 37 °C. With a Leica DM IL LED inverted fluorescence microscope, fluorescent pictures of 4–6 areas per well of 6 parallel wells were taken (all 43 pictures were taken with the following filters: excitation 450–490 nm, emission 500–550 nm for GFP; excitation 381–393 nm, emission 417–477 nm for Hoechst 33342). Pictures were evaluated by Cell Profiler 2.2.1 [17] yielding a transfection efficiency of 4.15 ± 1.86% (mean ± SD, *N* = 43). Despite low transfection efficiency, we processed the cells further without an enrichment step for transfected cells, but with very sensitive detection methods for recombinant protein (western blot for a 6xmyc tag), and PEDS1 activity (fluorescence precursor labeling of the cells and detection of fluorescent newly formed plasmalogen).

### Detection of recombinant protein as baseline for enzymatic activity calculations

To control for potential variations in the expression of transfected recombinant proteins, for every transfection the amount of recombinant protein was quantified in parallel wells by western blot using an anti-myc antibody staining the 6xmyc tag present on wildtype murine PEDS1 and on all murine PEDS1 mutants used (see below). Recombinant proteins were readily detectable for all transfection experiments using this method. Protein amount varied depending on the individual mutations and this was taken into account when quantifying the amount of plasmalogens formed (see below).

### Fluorescent labeling of newly formed plasmalogens in transfected cells

Cells were fed for further 24 h with 5 µM 1-*O-*pyrenedecyl-*sn*-glycerol (Otava Ltd, Vaughan, Ontario, Canada). Cells were collected, washed once with phosphate buffered saline, immediately frozen in liquid nitrogen and stored at − 80 °C until analyzed. PEDS1-deficient HAP1 cells with transfected GFP included as controls were always completely free of newly synthesized fluorescent plasmalogens (for analysis see below). Due to the intense fluorescent signal of the pyrene label used, the detection limit of plasmalogen formation of the mutants was 0.1% of the wildtype controls. An aliquot (1/10) of the cells was collected separately for protein determination by the Bradford assay (Biorad, Feldkirchen, Germany) using bovine serum albumin as standard.

### Lipid extraction and analysis

Lipids were extracted twice with 500 µl chloroform/methanol 2:1 (v:v) with shaking 4 times 30 s and 20 Hz in a mixa mill (MM400, Retsch, Haan, Germany), addition of 100 µl of water, mixing and centrifugation for 10 min and 10,000×*g* at 4 °C. The organic phases were combined, dried and resuspended in 100 µl acetonitril/ethanol 1:1 (v:v). 10 µl of the extract was then treated either with 40 µl methanol/HCl (930 µl methanol + 70 µl 1 M HCl) to convert all plasmalogen-derived aldehydes as well as free aldehydes to the corresponding dimethyl acetals, or with 40 µl methanol/acetic acid (930 µl methanol + 70 µl 1 M acetic acid) to convert all free aldehydes to the corresponding dimethyl acetals as a control. The amount of the dimethyl acetals was then quantified by reversed phase HPLC and fluorescence detection (excitation 340 nm, emission 400 nm) by comparison of peak areas to external synthetic standards of pyrenedecanoic acid (Sigma).

### Quantification of recombinant protein expression by western blot

In parallel wells, cells were cultivated after transfection for 48 h, collected, suspended in 200 µl 0.1 M Tris.HCl buffer, pH 7.6 containing 0.25 M sucrose, and protein content was determined by the Bradford assay with serum albumin as standard. 80 µg protein were then separated on 12.5% SDS gels, blotted to PVDF membranes (GE Healthcare, Vienna, Austria), incubated with a rabbit polyclonal anti-myc antibody (abcam ab9106), washed, incubated with a goat-anti-rabbit Cy5 labeled antibody (Fisher Scientific, Vienna, Austria), and the amount of fluorescence detected with a Typhoon 9410 laser scanner (GE Healthcare, excitation 630 nm, emission 670 nm).

### Calculation of results relative to protein expression and to wildtype controls

Each mutation was tested in three independent experiments, and transfections with myc-tagged wildtype plasmids were included as control in all transfections and western blot experiments. Also included in all experiments were negative controls using transfection of a green fluorescence protein expressing plasmid pEGFP-N1 (Clontech). For every single data point, the amount of labeled vinyl ether bond formed per cellular protein was related to the amount of recombinant protein detected and expressed in % relative to the wildtype control which was always run in parallel for each replicate.

### Homology modelling of murine PEDS1

Homology modelling was performed by using MOE (Molecular Operating Environment, version 2020.09, Molecular Computing Group Inc., Montreal, Canada). To avoid perturbations by freely charged functional groups, we capped the C-terminal and N-terminal parts of each domain with acetylamide (ACE) and *N*-methylamide for the following minimization and simulation runs. We modeled the position of the iron ions based on the template structure (PDB accession code: 6WF2) and relaxed their position by a minimization cycle. However, the exact position of the iron ions still remains rather speculative and might even change as part of the reaction mechanism. The starting structure for the simulation was prepared in MOE using the Protonate 3D tool. To neutralize the charges in our simulation box, we used the uniform background charge. Using the tleap tool of the AmberTools20 package, the crystal structures were soaked in a cubic water box of TIP3P water molecules with a minimum wall distance of 10 Å to the protein. For the structure model, parameters of the AMBER force field 14SB were used. The structure was carefully equilibrated using a multistep equilibration protocol. Then MD simulations were performed for 200 ns in an NpT ensemble using pmemd.cuda. Bonds involving hydrogen atoms were restrained by applying the SHAKE algorithm, allowing a time step of 2.0 fs. Atmospheric pressure (1 bar) of the system was set by weak coupling to an external bath using the Berendsen algorithm. The Langevin thermostat was used to maintain the temperature during simulations at 300 K. Additionally, the PEDS1 structure was predicted using the machine learning approach AlphaFold2 (v2.0) (https://github.com/deepmind/alphafold). The AlphaFold2 network directly predicted the 3D coordinates of all heavy atoms for the PEDS1 protein, by just using the primary amino acid sequence. The obtained structures were refined including AMBER-relax option to enhance the accuracy of the side chain geometries.

## Results

### Importance of conserved amino acids for enzymatic activity

**Fig. 2:**
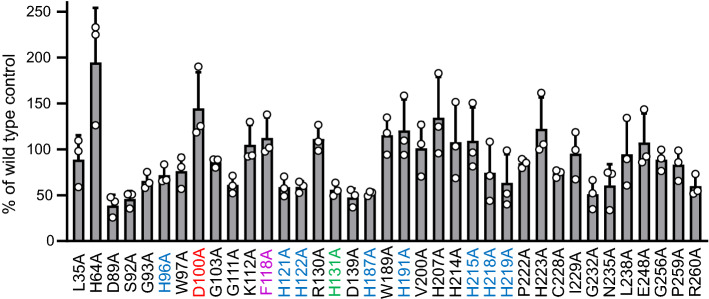
Expression of recombinant proteins in PEDS1-deficient HAP1 cells. C-terminally 6xmyc tagged murine PEDS1 expression plasmids carrying the indicated mutations were transiently transfected to PEDS1-deficient HAP1 cells and the amounts of recombinant proteins in comparison to an expression plasmid carrying the wildtype open reading frame were monitored by western blot as detailed in the “Materials and methods” section. Results show mean ± SD for three independent experiments in percent relative to the respective controls using the wildtype plasmid. The color code is identical to Fig. 1

**Fig. 3:**
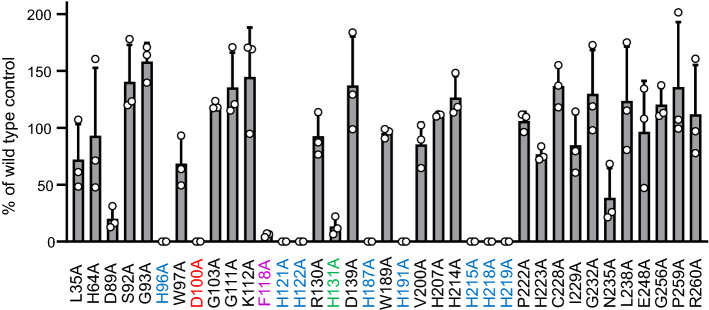
Formation of labeled plasmalogen per recombinant protein expression. C-terminally 6xmyc tagged murine PEDS1 expression plasmids carrying the indicated mutations were transiently transfected to PEDS1-deficient HAP1 cells. After 24 h, cells were fed with 1-*O*-pyrenedecyl-*sn-*glycerol for another 24 h, the cells harvested, lipids extracted and the amount of pyrene-labeled plasmalogen determined. All values were related to the amount of recombinant protein expressed as detailed in the “Materials and methods” section. Results show mean ± SD for three independent experiments in percent relative to the respective controls using the wildtype plasmid. The color code is identical to Fig. 1

In previous work, it was shown that the fully conserved histidines 96, 121, 122, 187, 191, 215, 218 and 219 in murine PEDS1 and the corresponding residues in CarF and human PEDS1 are essential for PEDS1 enzymatic activity (labeled blue in the figures) [2, 3]. Exchange of histidine 131 to alanine which produced inactive mutants in CarF and human PEDS1/TMEM189 in a light response assay in *M. xanthus* [2] had a residual activity of 7.4 ± 3.5% as compared to wildtype controls in the HPLC/fluorescence assay [labeled green (mean ± SD, *N* = 3)] [3]. This residual activity can structurally be explained, as H131 is not located within the di-iron binding site, but actually lies behind H121, which directly interacts with the iron ions. The partial loss of activity might result from the interaction of H131 with H121, indirectly influencing the iron binding site. In the present work, we mutated all other fully conserved amino acids (Fig. 1) each to alanine and tested the impact on the formation of pyrene-labeled 1-*O*-alk-1′-enyl lipids from fed 1-*O*-pyrenedecyl-*sn*-glycerol in PEDS1-deficient HAP1 cells. After transfection of the various mutated expression clones, the amount of recombinant protein expressed varied from 38.8 ± 9.5% to 194.7 ± 48.7% (mean ± SD, *N* = 3) of wildtype, depending on the specific mutation (Fig. 2). Nevertheless, recombinant protein could be readily detected and quantified in all experiments for all mutants. All amounts of vinyl ether concentrations were related to the amount of recombinant protein detected, and compared to the wildtype control (see “Materials and methods” for details). Figure 3 summarizes the effects of the replacement of the conserved amino acids to alanine. Surprisingly, only the mutation of D100 (labeled red) led to a total loss of enzymatic activity (less than 0.1% of wildtype). Clear attenuation of labeled vinyl ether bond formation was also observed for F118A (5.9 ± 1.4%, labeled magenta in the Figures). All other mutated plasmids lead to more than 20% formation of the vinyl ether bond as compared to wildtype plasmid (Fig. 3).

### A structural model for murine PEDS1

**Fig. 4:**
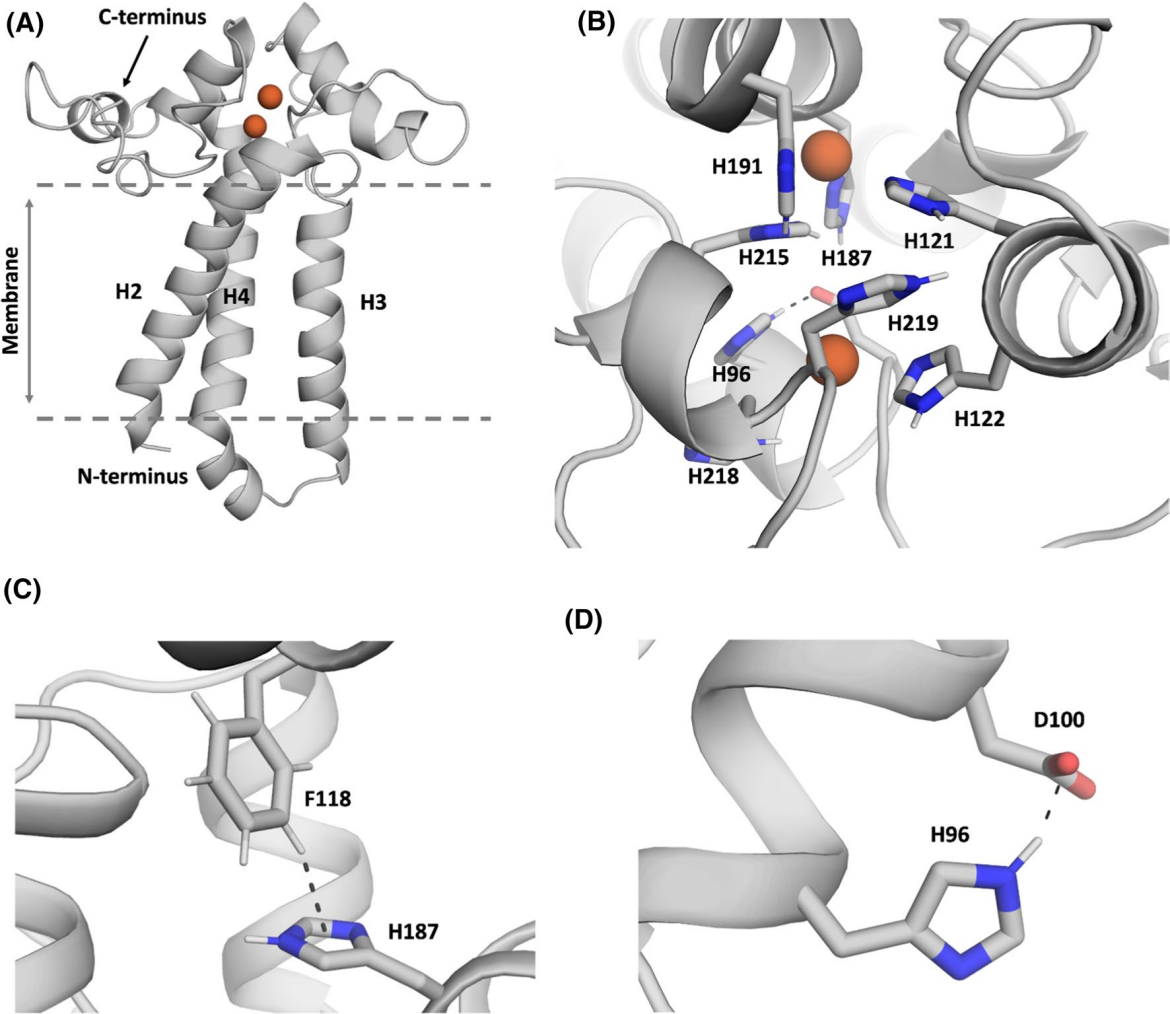
A predicted structure for murine PEDS1 based on homology modeling to SCD1. **A** Side view of the whole structure model of the PEDS1 with the two iron ions bound. **B** Top view of the structure model of the PEDS1, focusing on the histidine residues, which stabilize and directly interact with the di-iron center. **C** Amino acid residue D100 forms a hydrogen bond with residue H96 and contributes to stabilizing the iron ion. **D** Residue F118 makes a T-stacking interaction with H187 thereby stabilizing the iron ion and the protein conformation

**Fig. 5:**
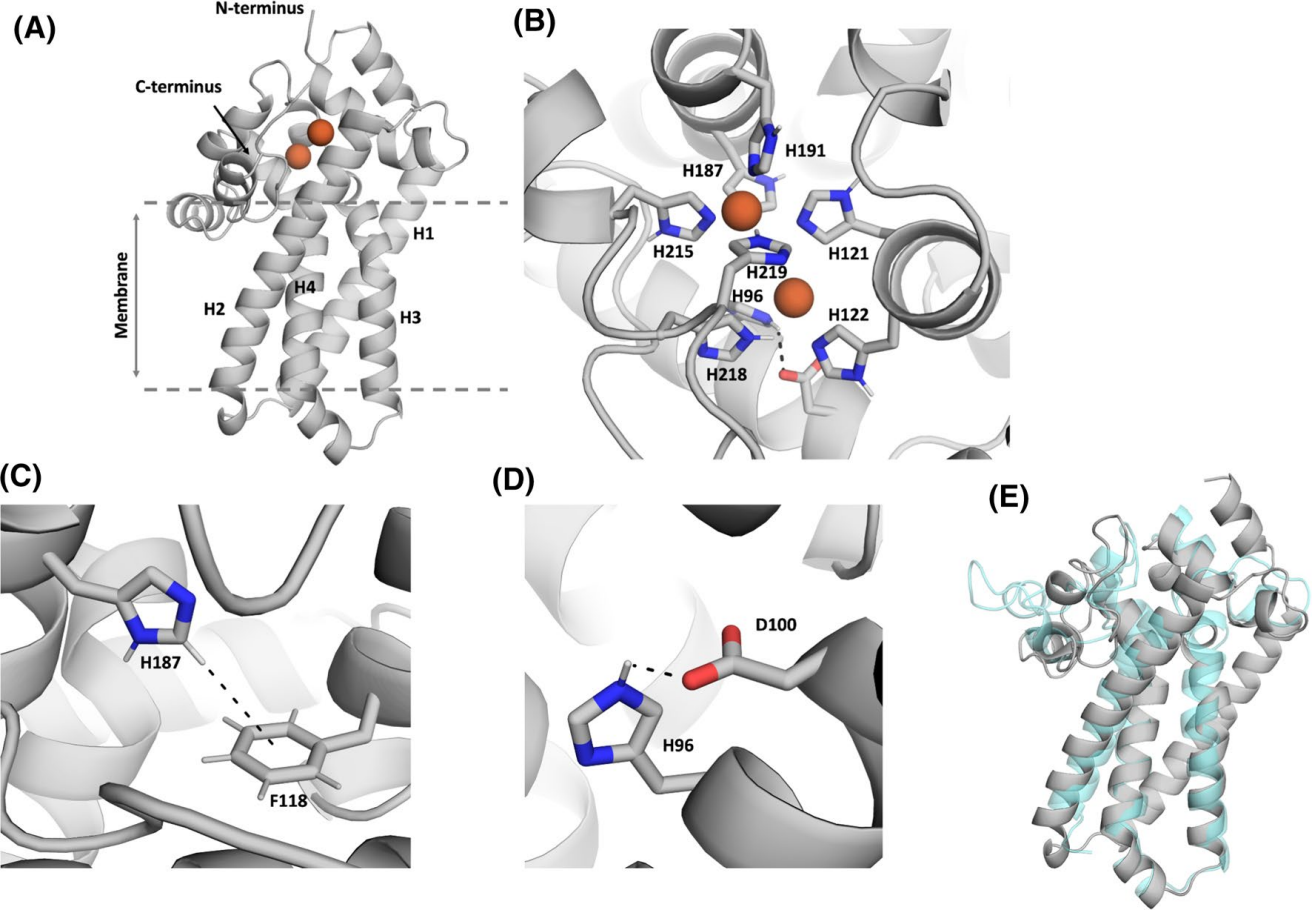
Structure prediction for the murine PEDS1 by using **“**AlphaFold2”. **A** Side view of the whole structure model of the PEDS1 with the two iron ions bound. Parts of the N-terminus and the first transmembrane helix are shown in the **“**AlphaFold2” model (residues 23–73), which have not been modeled in Fig. 4A. **B** Top view of the structure model of the PEDS1, focusing on the histidine residues, which stabilize and directly interact with the di-iron center. **C** In line with the observation in Fig. 4C, amino acid residue D100 forms a hydrogen bond with residue H96 in the “AlphaFold2” model and thereby contributes to stabilizing the iron ion. **D** Residue H187 makes a T-stacking interaction with F118 thereby stabilizing the iron ion and the protein conformation. **E** Structural overlay of the homology model (cyan) and the “AlphaFold2” model (gray) showing their overall similarity

We predicted the structure of PEDS1 by building a homology model based on the X-ray structures of the stearoyl-CoA desaturase 1 (SCD1) and the Scs7p hydroxylase (PDB accession codes: 6WF2 and 4ZR0, respectively). As the sequence alignment for the first transmembrane helix was really low and the first part of the model was highly unreliable, we only considered three transmembrane helices in our model and omitted the parts of the N-terminus (residues 1–22) and the first transmembrane helix (residues 23–73). The obtained structure model was then carefully equilibrated using a multistep equilibration protocol. Despite the low sequence identity of < 20%, the model predicted a structure of PEDS1 resembling that of SCD1 in that extensive alpha helices extended essentially in one direction from the catalytic center which we identified in our alignment by conserved histidine residue positions and cation-binding sites (Fig. 4A). Reassuringly, the model with the lowest energy predicted those histidines to bind the two metal ions which are fully conserved and which had previously been experimentally shown to be essential for the enzymatic activity ([2, 3], Fig. 4B). Amino acid residue D100 formed a hydrogen bond with residue H96 and contributed to stabilizing the iron ion (Fig. 4C). We then set up a 200 ns molecular dynamics simulation of the homology model to relax the amino acid orientations and to obtain a probability of the D100–H96 interaction. We found that this hydrogen bond interaction was present in 78% of the frames. In the other 22% of the frames the residue D100 formed a hydrogen bond with H187 or directly interacted with the iron ion. Amino acid residue F118 made a T-stacking interaction with H187 and thereby stabilized its conformation, ensuring a stabilization of the iron ion (Fig. 4D). Recently, a novel machine learning approach, “AlphaFold2” revolutionized protein structure prediction [18]. “AlphaFold2” is a machine learning approach, that incorporates both physical and biological knowledge about the protein structure. Thus, we also predicted the PEDS1 structure by using “AlphaFold2”. Figure 5 shows the modeled PEDS1 structure, highlighting the catalytic histidine residues, as well as the key interactions of D100 with H96 and H187 with F118. The predicted interactions and their respective interaction partners in Fig. 4C, D were also present in the new “AlphaFold2” model. By comparing our homology model with the “AlphaFold2” model, we found very high overall structural similarity (Cα-RMSD: 3.2 Å). The biggest divergence between the two models was the prediction of the loop ranging from residue 197 to residue 213. Additionally, the “AlphaFold2” model also contained parts of the N-terminus and the first transmembrane helix (residues 23–73), which could not be modeled reliably with the homology model.

## Discussion

As revealed by the analysis of tissues of PEDS1-deficient mice [3], the *Peds1*(*Tmem189*) gene is the only gene in mice capable of introducing the vinyl ether bond into plasmalogens, thereby providing a lipid class with special chemical and biochemical properties. The physiological role of plasmalogens is topic of ongoing research and has just starting to be understood. The recent identification of the gene encoding PEDS1 [2–4] enabled investigation of residues important for enzymatic activity by site-directed mutagenesis in recombinant expression systems. While the role of conserved histidines had already been investigated in the course of the identification of the gene coding for PEDS1 [2, 3], the role of other conserved amino acid residues was still unknown. Here, we demonstrate that among 20 further totally conserved amino acid residues (Fig. 1) only 1, aspartate 100, was absolutely essential for enzymatic activity. A further residue, phenylalanine 118, left only 6% residual activity when mutated. All other conserved residues appeared to be less important for enzymatic activity (Fig. 3).

PEDS1 is a very labile integral membrane protein. A purification strategy for the PEDS1 homolog CarF from *M. xanthus* was recently published [2]. The enzyme had partial activity and bound approximately two equivalents of iron [2]. However, nobody so far succeeded in purification of an highly active form and crystallizing it, a drawback it shares with other membrane-bound desaturases and hydroxylases which are characterized by a conserved eight-histidine-motif [19]. Notable exceptions are mammalian stearoyl-CoA desaturase SCD1 [11, 12, 15] and fatty acid alpha hydroxylase from yeast [13], which have been crystallized and the structure determined. To interpret the results of our mutational analysis, we, therefore, pursued to generate a homology model, despite the limited homology of the amino acid sequences of PEDS1 with SCD1. Careful equilibration resulted in a model which predicted that the histidines previously found to be essential for enzymatic activity coordinated two iron atoms in a characteristic di-iron center [19]. In addition, the model could readily explain why aspartate 100 and phenylalanine 118 were so important for enzymatic activity. These two residues directly interact with one of the essential histidines each. These observations could be confirmed by predicting the structure with the recently published “AlphaFold2”. Even though the two models differ in a loop region, the positions of the histidines and the respective interactions are highly similar. Both resulting models of PEDS1 were monomers, as is the structure of SCD1, which we had used as a template. In gel filtration experiments, however, the *M. xanthus* homologue of PEDS1 eluted at approximately the size of a dimer [2]. Crosslinking experiments of recombinantly expressed SCD1 in Cos7 cells also suggested the presence of dimers and oligomers [20].

In contrast to aspartate 100 and phenylalanine 118, the further 18 of the 20 conserved amino acids investigated in the present work, surprisingly showed no or only minor impact on enzymatic activity. One possibility is that their exchange of a single residue is not sufficient to impact on the overall structural requirements for the enzyme. Additionally, we found a pattern in the structure showing that the conserved histidine residues not only in the models for PEDS1 but also in the structure of the SCD1 have an essential glutamate, aspartate or glutamine residue in close proximity, which can contribute to stabilize the di-iron-binding site.

## Data Availability

The original data presented in the study are included in the article, further inquiries can be directed to the corresponding author.
